# Autonomous buckling of micrometer-sized lipid-protein membrane patches constructed by *Dictyostelium discoideum*

**DOI:** 10.1186/1754-1611-9-3

**Published:** 2015-01-21

**Authors:** Kei Takahashi, Taro Toyota

**Affiliations:** Department of Basic Science, Graduate School of Arts and Sciences, The University of Tokyo, 3-8-1 Komaba, Meguro, Tokyo, 153-8902 Japan; Research Center for Complex Systems Biology, Graduate School of Arts and Sciences, The University of Tokyo, 3-8-1 Komaba, Meguro, Tokyo, 153-8902 Japan

**Keywords:** Substrate-supported lipid membrane, Spin coating, Phosphatidylcholine, Phosphatidylinositides, *Dictyostelium discoideum*

## Abstract

**Background:**

The cytosol of amoeba cells controls the membrane deformation during their motion *in vivo*. To investigate such ability of the cytosol of amoeba cell, *Dictyostelium discoideum* (*Dictyostelium*), *in vitro*, we used lipids extracted from *Dictyostelium* and commercially available phospholipids, and prepared substrate-supported lipid membrane patches on the micrometer scale by spin coating.

**Results:**

We found that the spin coater holder, which has pores (pore size = 3.1 mm) of negative pressure to hold the cover glass induced the concave surface of the cover glass. The membrane lipid patches were formed at each position in the vicinity of the holder pores and their sizes were in the range of 2.7 to 3.2 × 10^4^ μm^2^. After addition of the cytosol extracted from *Dictyostelium* to the lipid membrane patches, through time-lapse observation with a confocal laser scanning fluorescence microscope, we observed an autonomous buckling of the *Dictyostelium* lipid patches and localized behaviours of proteins found within.

**Conclusion:**

The current method serves as the novel technique for the preparation of film patches in which the positions of patches are controlled by the holder pores without fabricating, modifying, and arranging the chemical properties of the solution components of lipids. The findings imply that lipid-binding proteins in the cytosol were adsorbed and accumulated within the *Dictyostelium* lipid patches, inducing the transformation of the cell-sized patch.

**Electronic supplementary material:**

The online version of this article (doi:10.1186/1754-1611-9-3) contains supplementary material, which is available to authorized users.

## Background

Micrometer-sized biochemical machinery has garnered much attention as an energy conversion device that converts chemical energy to kinetic energy and as a model of cell machinery that minimizes energy consumption [1–3]. The research trend on biochemical machinery has contributed to supramolecular chemistry and soft matter physics for understanding cell dynamics. Model construction for cell dynamics, such as membrane buckling [4, 5], has attracted the attention of researchers because such cell dynamics are closely related to amoeba motion which highly functionalized with the coupling of cellular mechanics and sensors [6]. Conventionally, the buckling of a cell membrane is examined by using a narrow glass capillary with the inner diameter of several micrometers. The aspiration through the glass capillary attached on the cell membrane induces locally the cell membrane deformation (and even the transformation of the whole cell body). In this study, we focus on the membrane deformation controlled by its cytosol in amoeba motion *in vitro*. The *in vitro* self-organization of lipid membranes and proteins in a micrometer-sized reaction surface or two-dimensional plane is important. For example, essential combinations of proteins associated with membrane deformation [7, 8] or cell division cycle [9] can generate a pattern formation and time course changes of a model cell membrane supported on a substrate. Our challenge contains the problem that the parameter space composed of the lipids and proteins related to amoeba motion is too vast to investigate often arises because each component should be completely purified and mixed in a sequential manner. Here, we overcame this obstacle by using lipids and cytosol extracted from an amoeba cell and constructing micrometer-sized substrate-contacting lipid-protein membrane patches. The constructed system enables us to not only examine the essence of the membrane-assisted biochemical machinery of amoeba motion without the cell complexity, but also to avoid the practical difficulty of incomplete encapsulation of cytosol extracts in the case of vesicle-type biochemical machinery.

**Figure 1:**
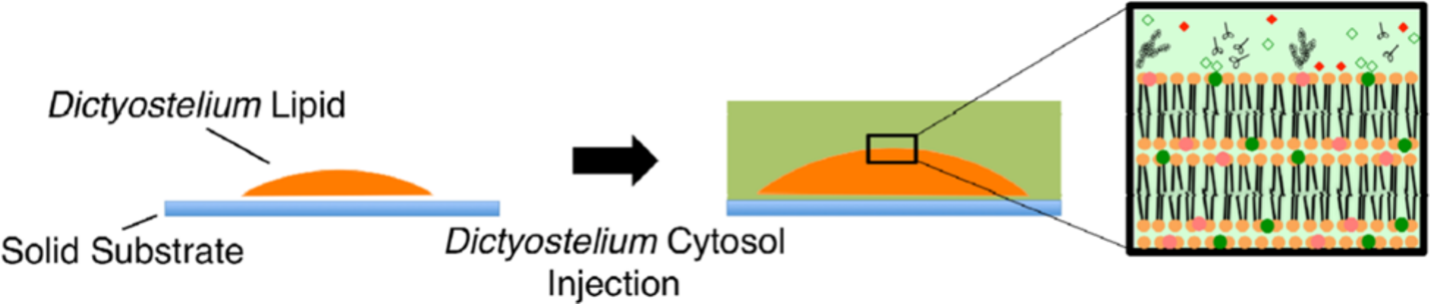
**Schematic illustration of the lipid patch buckling induced by the cytoplasmic extract injection.** Both the lipids and a cytosolic extract are extracted from *Dictyostelium*.

The current purpose is thus to investigate the ability of the cytosolic extract of amoeba cells for deformation of their lipid membrane film *in vitro* (Figure 1). First, we focus on the cytosol and lipid extracts from *Dictyostelium discoideum* (*Dictyostelium*), because *Dictyostelium* is a type of amoeba that sprightly shows amoeba motion at room temperature (20 – 23°C). The experiment can be undergone without heating or cooling from room temperature. Second, we adopt the spin coating method to prepare a lipid membrane film constructed on a substrate because lipid mixtures extracted from living cells can hardly form well-defined membrane structures, such as single bilayer membranes, supported on a substrate. The spin coating method produces micrometer-sized patches with a thickness of several micrometer when the membrane-forming components are heterogeneous, resulting from the interaction between the organic solvent and the solutes [10]. Third, in order to trace the transformation of such lipid membrane film in a three-dimensional manner after the addition of the cytosolic extract, we performed time lapse observations using a confocal laser scanning fluorescence microscope.

## Results

### Micrometer-sized patches of lipid membrane film prepared by spin coating

**Figure 2:**
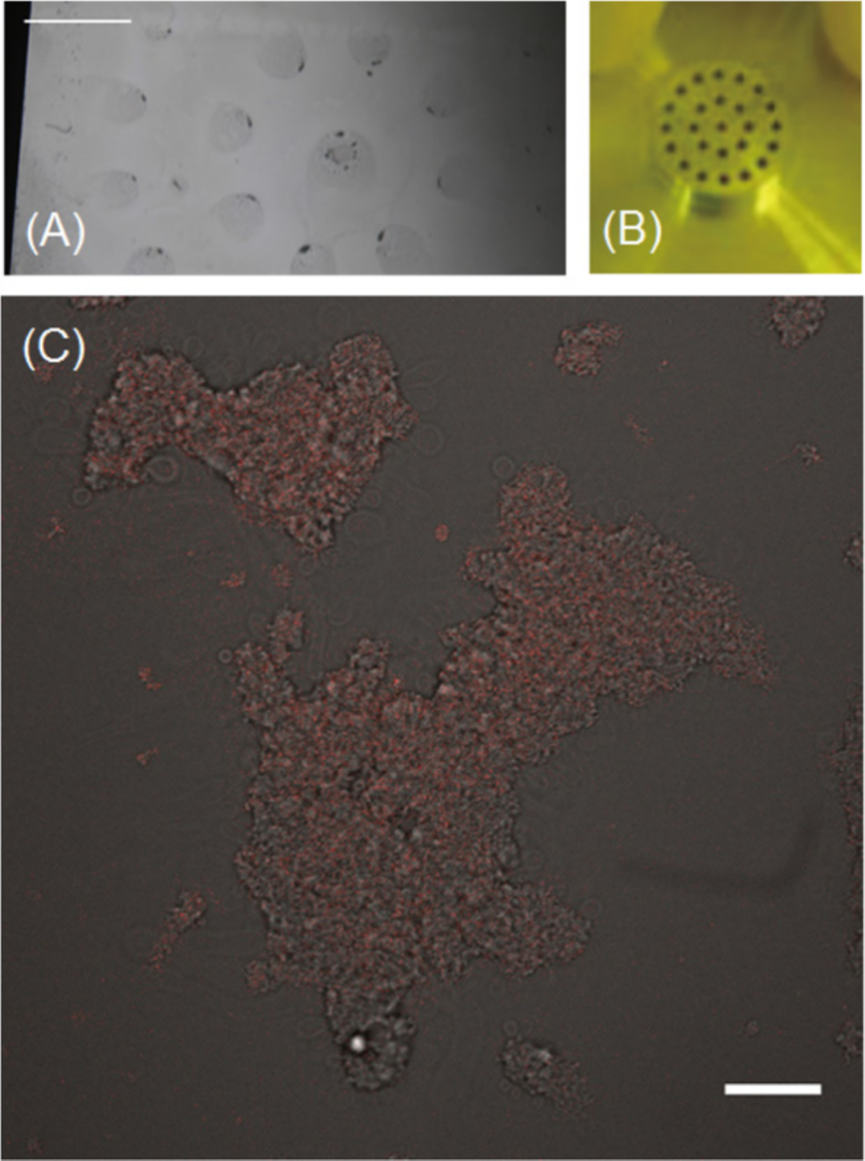
**Microscopy images of the membrane lipid patch. (A)** Bright field microscopy image of the cover glass with *Dictyostelium* lipid membrane film prepared by spin coating (bar = 3 mm). **(B)** Photograph of the cover glass holder. **(C)** Reconstructed image of the bright field microscopy image and the autofluorescence images of the typical patch of *Dictyostelium* lipid membrane film (bar = 30 μm).

The spin coating method for the lipid membrane film supported on a glass substrate conventionally enables us to prepare heterogeneous films using a lipid mixture [10]. After extraction of the lipid mixture from the wild-type *Dictyostelium*, AX4 cells, we observed the lipid membrane film of *Dictyostelium* prepared by the spin coating method on the millimeter scale by light microscopy. We found a lipid membrane film pattern on the cover glass (Figure 2A), which was held by the aspiration pores of the spin coat holder (Figure 2B). The lipid membrane film pattern was consisted of 25 islands (approximately 2.5 - 3 mm in diameter) which contained several patches on the micrometer scale (black spots shown in Figure 2A). In order to visualize such patches precisely, we obtained both the bright field microscopy images and the autofluorescence images of the lipid membrane film by using confocal laser scanning fluorescence microscopy. Two sets of autofluorescence images were obtained by using two irradiation lasers and the corresponding band-pass filter and dichroic mirror units (see Methods). Figure 2C shows the reconstructed images of the bright field microscopy and autofluorescence images of one of the patches. The regions between the islands were also covered with very thin lipid membranes (data not shown). Judging from the image analysis and taking into account the diffraction limit of light and the special resolution, the sizes of the patches were in the range of 2.7 to 3.2 × 10^4^ μm^2^ and the size range was observed reproducibly.

As a reference experiment, when we dropped a chloroform solution of 1-palmitoyl-2-oleoyl-3-*sn*-glycero-3-phosphocholine (POPC) and 10 mol% of 1,2-hexadecanoyl-3-*sn*-glycerophosphatidylinositol-(3,4,5)-trisphosphate (PIP3), islands and lipid membrane patches were also formed at the positions of the holder pores after solvent evaporation (see Additional file 1: Figure S1). Based on the micrograph of the patches, the contrast of the patches was stronger than that of the thin film island, indicating that the lamellarity of the patches was greater than that of the thin film. It implies that the patches composed of PIP3 and POPC grew in a thin layer of the organic solution which covered the concave surface after spin coating. Therefore, we could establish the preparation method of film patches in which the positions of patches are controlled in both cases of natural crude lipids and purified lipids. We thus focused on the patches by observing their deformation or the construction of micrometer-scale amoeba motion model.

### Buckling of lipid membrane film after injection of cytosol extract

**Figure 3:**
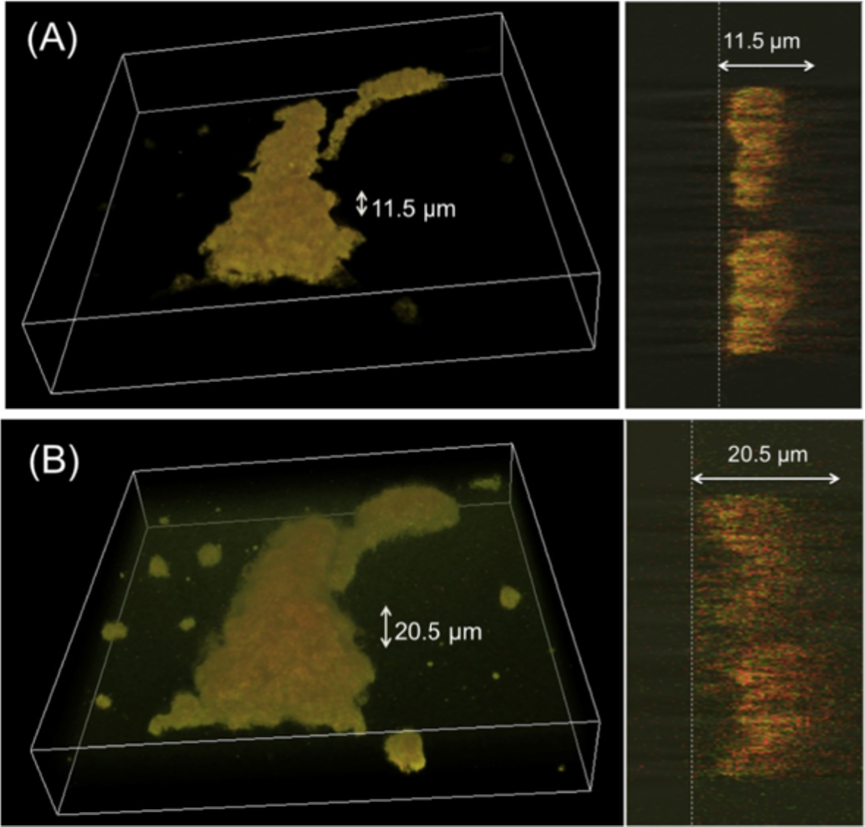
**Microscopy image of the buckled** ***Dictyostelium*** **lipid patch after injection of the cytosol extract of** ***Dictyostelium*** **cells.** 3D-merged reconstructed images of the autofluorescence images of a typical *Dictyostelium* lipid patch obtained by confocal laser scanning fluorescence microscopy with both Red and Green detection modes before **(A)** and 30 min after **(B)** injection of cytosol extract. The volume size of observation space is 210 μm x 210 μm x 37.5 μm. Each x-z plane cross-section of the 3D merged reconstructed image was attached in the right column. Dashed lines correspond to the surface of the glass slide. The height indicates the distance between the surface of the glass slide and the peak top position of the autofluorescence images.

**Figure 4:**
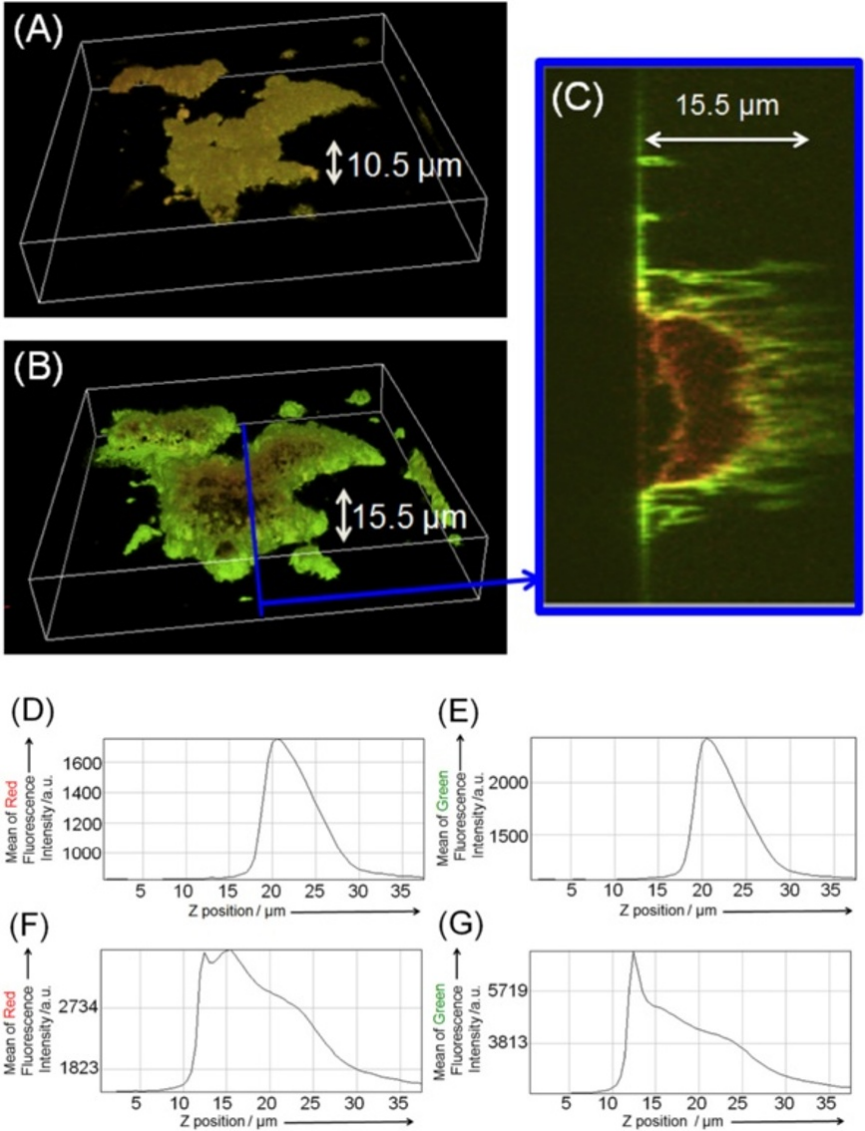
**Microscopy image of the buckled** ***Dictyostelium*** **lipid patch after injection of the cytosol extract of PH-crac-RFP/PTEN-GFP co-expressing cells.** 3D merge of reconstructed fluorescence images of a typical patch of the *Dictyostelium* lipid membrane film obtained by confocal laser scanning fluorescence microscopy, with both Red and Green fluorescence filter units, before injection of cytosol extract. **(A)** 3D merge of reconstructed fluorescence images of a typical patch of the *Dictyostelium* lipid membrane film obtained by confocal laser scanning fluorescence microscopy, with both Red and Green fluorescence filter units, before injection of cytosol extract. The volume size of observation space is 210 μm x 210 μm x 37.5 μm. The mean fluorescence intensity of each Red and Green fluorescence image was plotted along the z position in **(D)** and **(**E**)** respectively. **(B)** 3D merge of reconstructed fluorescence images and **(C)** the cross-section reconstructed fluorescence images of a typical patch of the lipid membrane film obtained by confocal laser scanning fluorescence microscopy with both Red and Green fluorescence filter units, 30 min after injection of the cytosol extract of PH-crac-RFP/PTEN-GFP co-expressing cells. The mean fluorescence intensity of each Red and Green fluorescence image was plotted along z position in **(F)** and **(G)**, respectively. The height shown in **(A-C)** indicates the distance between the surface of the glass slide and the peak top position of the autofluorescence images.

After setting up a chamber attaching the cover glass to a dish by a double sided tape, we injected the cytosol (25 μL) extracted from AX4 cells. As shown in Figure 3, the buckling of the lipid film patch was traced immediately after wetting the *Dictyostelium* lipid patch by PBS and following injection of the AX4 cytosol (see Additional file 2: Video 1). The *Dictyostelium* lipid patch exhibited autonomous transformation for the initial 10 min, resulting in the swelling in the x-y plane. Moreover, we observed a mesh-like structure after the transformation in the bright field microscopy images. Even though we observed the formation of vesicles, the diameter of which were several micrometer at the path of lipid membrane film immediately after the pre-wetting and the injection of cytosol, it should be noted that the cytosol induced the patch buckling and the transformation of the dome-like shape, which is several tens of micrometers in diameter. Moreover, the cytosol of genetically modified *Dictyostelium* cells, containing the Pleckstrin homology domain of cytosolic regulator of adenylyl cyclase (PH-crac) tagged with RFP (PH-crac-RFP) and the phosphatase and tensin homolog (PTEN) tagged GFP (PTEN-GFP) [11], was injected. The initial lipid membrane, with a maximum height of 10.5 μm on average (Figure 4A) under treatment with PBS for 1 min for pre-wetting started to buckle after 10 min in the chamber. In 25 min, *Dictyostelium* lipid patch stopped buckling and an equilibrium state was reached 30 min after *Dictyostelium* cytosol injection into the chamber (see Additional file 3: Video 2 and Additional file 4: Video 3). The average maximum height of lipid patches was 15.5 μm as seen in 3D reconstructed images. Figure 4B shows that PH-crac-RFP and PTEN-GFP localized on micrometer-sized patch of lipid membrane, were mutually exclusive. In particular, GFP fluorescence was enriched at the edge of the patch and surrounded the RFP-fluorescing regions inside the patch (Figure 4C). Taking into account the fluorescence intensity of the confocal fluorescence images of the patch at each z-position (interval = 0.5 μm), we evaluated the change in the width and height of the patch before and 30 min after cytosol injection. By measuring the fluorescence intensity profile along the z-position in both Red and Green modes (Figures 4D,E,F, and G), we found that the height of the patch increased 30 min after cytosol injection (Figures 4E,G). The maximum thickness of the patch increased by a factor of 2.1 ± 0.3 after cytosol injection, indicating that membrane deformation and buckling dynamics took place after cytosol injection.

**Figure 5:**
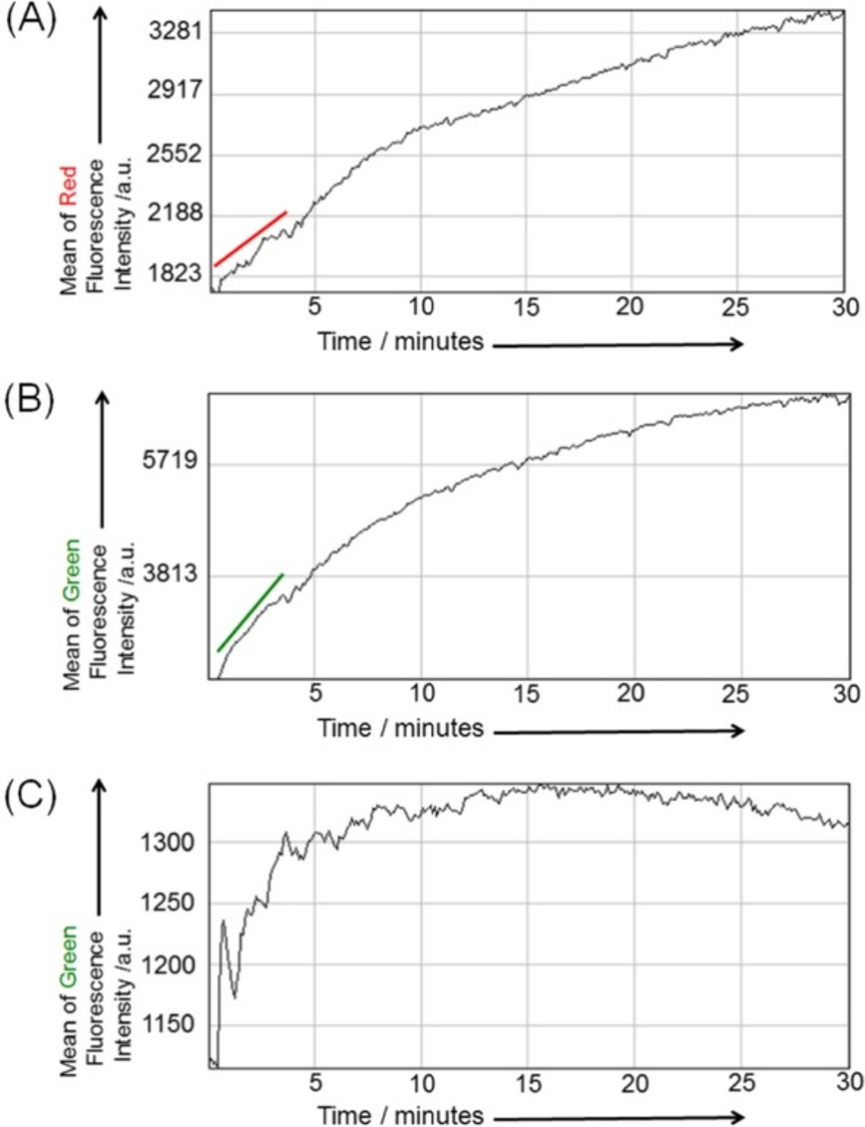
**Time course change of fluorescence intensity of the** ***Dictyostelium*** **lipid patch.** Time course change of the mean fluorescence intensity analyzed by fluorescence images (z position; two image frames (~1 μm) above the glass slide) of the *Dictyostelium* lipid patch obtained by confocal laser scanning fluorescence microscopy images with both Red **(A)** and Green **(B)** fluorescence detection filter units after injection of the cytosol extract of PH-crac-RFP/PTEN-GFP co-expressing cells. The slope in the range of 30 – 180 s is depicted in each diagram. **(C)** Time course change of the mean fluorescence intensity analyzed by fluorescence images (z position; two image frames (~1 μm) above the glass slide) of a typical patch of the POPC/PIP3 membrane film obtained by confocal laser scanning fluorescence microscopy images with Green fluorescence detection filter units after injection of the cytosol extract of PH-crac-RFP/PTEN-GFP co-expressing cells.

Then, the time course changes in the fluorescence intensity of *Dictyostelium* lipid patches in Red and Green modes were analyzed cytosols containing PH-crac-RFP and PTEN-GFP. The mean fluorescence intensity of Green and Red modes of all pixels on each image frame (Red mode, Figure 5A; Green mode, Figure 5B) increased immediately after cytosol injection (*t* = 30 s). When we compared the initial increase in fluorescence intensity in the range of 30 - 180 s, we found that PTEN-GFP was localized faster than PH-crac-RFP. As a reference experiment, we injected *Dictyostelium* cytosol containing PTEN-GFP and PH-crac-RFP to the chamber of lipid patches composed of a POPC/PIP3 membrane, and the fluorescence image obtained in the same procedure showed that PTEN-GFP localized on the patches and its localization speed was similar to that observed in the case of *Dictyostelium* lipid patch (Figure 5C). Because the lipid patch of POPC/PIP3 was stained with TexasRed-DHPE, we omitted the change in Red fluorescence in this reference experiment.

In another reference experiment, we injected *Dictyostelium* cytosol from PH-crac-RFP/PTEN-GFP co-expressing cells treated at 65°C for 1 h into the chamber of *Dictyostelium* lipid patches. Additional file 5: Figure S2 in Supporting Information shows the autofluorescence images of the *Dictyostelium* lipid patch before and 30 min after cytosol injection. Analyzed by time course change of the mean fluorescence intensity (z position; two image frames (~1 μm) above the glass slide), GFP fluorescence increased and the edge of each patch fluoresced 10 min after cytosol injection. Moreover, PTEN-GFP localization in the patch occurred but membrane thickness did not increase. When we injected the cytosol, which had been exposed to either autoclave processing or phosphate buffer treatment into the *Dictyostelium* lipid patch, no patch deformation was observed in either case. The POPC supported membrane on the cover glass remained unaltered after injection of cytosol of *Dictyostelium* cytosol from PH-crac-RFP/PTEN-GFP co-expressing cells.

**Figure 6:**
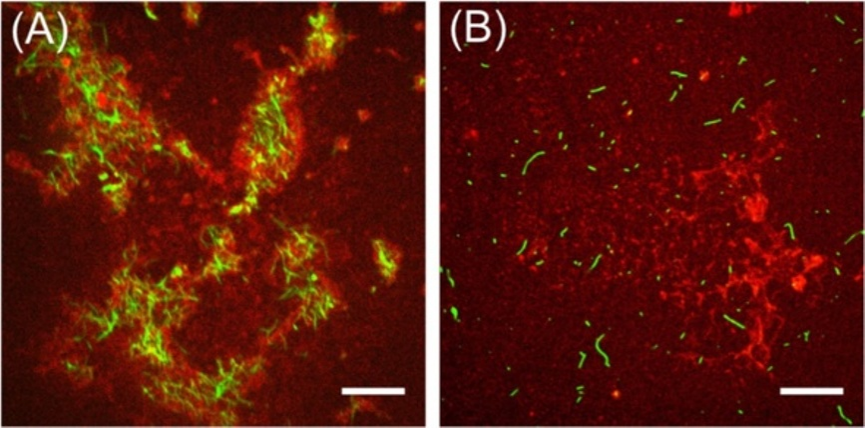
**Microscopy images of F-actin stained by phalloidin on the membrane lipid patches.** Confocal laser scanning fluorescence microscopy images of the *Dictyostelium* lipid patch **(A)** and POPC membrane film **(B)** after cytosol extract injection and following phalloidin staining (bar = 10 μm).

It is known that F-actin polymerizes on *Dictyostelium* lipid membranes as a result of the downstream chemical reaction network of phosphatidylinositides, known as PIP signalling [12]. We used phalloidin staining to visualize the localization of F-actin polymerization and bundling in the *Dictyostelium* lipid patch in the current study. After formalin fixing, the confocal laser scanning fluorescence microscopy images of the *Dictyostelium* lipid patches showed F-actin localization and polymerization in the patches (Figure 6A). No F-actin polymerization occurred on the POPC supported membrane (Figure 6B), indicating that the *Dictyostelium* lipid patch accumulated phosphatidylinositides.

## Discussion

### Lipid film patches prepared by spin coating

There are several methods for lipid membrane film preparations, such as casting [13], the spin coating [10, 14], Langmuir-Blodgett membrane formation [15–17], vesicle rupture [18–20], and water-in-oil droplet template methods [21]. The casting and spin coating methods are conventionally adopted in requesting membrane films composed of any kinds of lipids or mixtures. In the Langmuir-Blodgett membrane method, the formation of a lipid monolayer on a water surface is essential for the lipid molecular structure and the composition of lipid mixture. The vesicle rupture method relies on the stability of the lipid bilayer membrane for the formation of small unilamellar vesicles and giant unilamellar vesicles as the initial protocol step. The water-in-oil droplet template method is based on formation of stable water-in-oil droplets and the monolayers are stacked in layer-by-layer manner by using a microfluidic system. These last three methods have drawn much attention in terms of preparing well-defined bilayer membranes on a substrate. In the current study, the lipid mixture extracted from AX4 cells contains a various kinds of lipids as described later. In fact, previous reports on small unilamellar vesicles revealed that the components of each vesicle are dispersed even in one batch of the vesicle dispersion of lipid mixture [22, 23]. And the vesicle rupture methods in the conventional manners did not afford such lipid membrane film using the lipid mixture extracted from AX4 cells (data not shown). The water-in-oil droplet template method is hardly applied for the bilayer membrane formation of *Dictyostelium* because the organic solvent is optimized for not dissolving the lipid component but stabilizing the emulsion droplets. Besides, the casting method, where an organic lipid solution is dropped onto a substrate and the solvent is removed resulting in the multilayer membrane structure, also has the issue of regulating membrane formation under the evaporation of the solvent. Particularly, the control of lipid membrane film formation is likely difficult due to the wetting and de-wetting process of the solvent and the solubility of lipids. The spin coating method facilitates the preparation of lipid membrane films using lipids extracted from AX4 cells and allows the control of the organic solution thickness, dissolving lipids before solvent evaporation occurs and resulting in several types of lipid membrane films on the substrate. The micrometer-sized patches of organic thin film are also formed by the spin coating method by means of micro-fabricated surfaces [24, 25], chemically modified substrate surfaces [26, 27], and differences in solubility between each solute in the organic solution [28]. We found that the spin coater holder, which has pores (pore size = 3.1 mm) of negative pressure to hold the cover glass induced the concave surface of the cover glass and that the membrane film was formed at each position in the vicinity of the holder pores. As far as we know, this process may serve as the fourth technique for the preparation of film patches in which the positions of patches are controlled by the holder pores without fabricating, modifying, and arranging the chemical properties of the solution components. After spin coating, the concave surface of the cover glass was covered by a thin layer of the organic solution, and then the further evaporation of the solvent resulted in the island formation with large thin film at each concave surface. The diameter of the island was approximately 3 mm in the current study. The sizes of the lipid film patches at each island were in the range of 2.7 to 3.2 × 10^4^ μm^2^. This process resembles the patch formation from heterogeneous lipid solutions in the spin coating method [29–31].

As reported in previous articles [32–34], lipids extracted from *Dictyostelium discoideum* contain phosphatidylcholines, phosphatidylethanolamines, phosphatidic acids, and phosphatidylinositides. According to the thin layer chromatograms reported in these articles, phosphatidylcholines and phosphatidylethanolamines have a higher affinity to organic solvents such as chloroform compared to phosphatidic acids and phosphatidylinositides. These results suggest that the *Dictyostelium* lipid patches formed on the cover glass contain the core structure of low-solubility lipids (phosphatidic acids, phosphatidylinositides, and so on) and the film layers of high-solubility lipids (phosphatidylcholines, phosphatidylethanolamines, and so on). Moreover, the size of the patch is influenced by the initial step of *Dictyostelium* lipid extraction, namely the volume ratio of CHCl_3_/CH_3_OH (2:1, v/v) and the number of AX4 cells. We could systematically optimize the ratio as mentioned in the ‘Methods’ to obtain a patch size in the range of 2.7 to 3.2 × 10^4^ μm^2^. The thickness of the patches was 10.5 μm on average, judging by 3D constructed images of the autofluorescence images obtained by confocal laser scanning fluorescence microscopy.

### Autonomous buckling of the *Dictyostelium*lipid patch

The buckling of lipid membrane films to form micrometer-sized dome-like shapes from plain lamella is conventionally observed in the so-called electroformation method of giant vesicles [35, 36]. Under the application of an alternative voltage, after the injection of water or buffered solution into the lipid membrane film, the swelling process of the lipid membrane film is controlled by the electrophoresis of the membrane and the addition of water and water-soluble substances into the intermembrane space. Thus, the control of the external voltage and time interval can afford the formation of the dome-like and spherical shapes of giant unilamellar vesicles. In our study, the buckling of the lipid membrane film patch composed of *Dictyostelium* lipid autonomously took place under no external forces or stimuli after cytosol injection. We interpret the buckling mechanism of the lipid membrane film patch as follows.

The cytosol of *Dictyostelium discoideum* contains various types of membrane-binding proteins and/or lipid-conversion enzymes such as phospholipases, lipases, and phosphokinases. Since PH-crac and PTEN play important roles in membrane deformation during amoeba motion [37], we focused on their adsorbing process onto the *Dictyostelium* lipid patch by means of PTEN tagged by GFP (PTEN-GFP) and PH-crac tagged by RFP (PH-crac-RFP). As shown in Figure 5, in the initial stage immediately after cytosol injection, PTEN-GFP adsorbed more rapidly than PH-crac-RFP. Since PTEN-GFP adsorbed onto the POPC patch, which contained 10 mol% PIP3, it is implied that PTEN likely adsorbed onto the lipid membrane film. It is known that in living *Dictyostelium discoideum* cells, PTEN is one of the phosphokinases for phosphatidylinositides and tends to bind to the cell membrane during the resting mode of cells [38]. On the other hand, PH-crac tends to stay in the cytosol of resting cells and forms a lipid-protein complex with phosphatidylinositides when it adsorbs to the cell membrane [39, 40]. Because the amount of phosphatidylinositides is much smaller than that of other phospholipids in *Dictyostelium* lipid [41, 42], the initial adsorbing process of PH-crac on the *Dictyostelium* lipid patch is slower than that of PTEN. When the amount of adsorbed PH-crac gradually increases and the lipid-protein complex formation begins in the *Dictyostelium* lipid patch, it likely accumulates inside of the *Dictyostelium* lipid patch. This is probably because the phosphatidylinositides are organized primarily inside the patch and also, PH-crac competes with PTEN, which adsorbs and covers the *Dictyostelium* lipid patch to bind to the phosphatidylinositides. As a result, mutually exclusive distributions of PTEN-GFP and PH-crac-RFP were observed in the buckled *Dictyostelium* lipid patch. The *Dictyostelium* lipid patch is then buckled and grows to become a dome-like shape because of unbalanced osmotic pressure. As reference experiments, when we injected a cytosol, which had been treated at 65°C for 1 h just before use, we observed an increase in the fluorescence intensity of PTEN-GFP in the *Dictyostelium* lipid patch but no buckling (Additional file 5: Figure S2). This indicates that only the adsorbed PTEN on the patch had no potential to induce buckling. After treating the cell culture with LY294002 before the extraction, we injected the extracted cytosol to the *Dictyostelium* lipid patch and observed the buckling and dome-type shape of the patch. The result implied that the activity of phosphatidylinositol-3-kinase (PI3K), which is one of the phosphokinases for phosphatidylinositides and is inhibited by LY294002, exerts less influence on membrane buckling than PTEN and PH-crac. In order to clarify the phosphatidylinositides accumulation in the *Dictyostelium* lipid patch, we performed phalloidin staining for F-actin polymerization and bundle formation in the buckled *Dictyostelium* lipid patch because these chemical reactions occur at the sites of phosphatidylinositides aggregation accumulated in the *Dictyostelium discoideum* cell membrane [33]. Since bundles of polymerized F-actin were observed in the *Dictyostelium* lipid patch, it was confirmed that the *Dictyostelium* lipid patch contains phosphatidylinositides after cytosol injection. It implies that, among the phosphatidylinositides, 1,2-dialkyl-3-*sn*-glycerophosphatidylinositol-(4,5)-diphosphate, which is the product from 1,2-dialkyl-3-*sn*-glycerophosphatidylinositol-(3,4,5)-triphosphate by PI3K, is less effective than PIP3 for the PTEN-GFP adsorbing behaviour and PH-crac-RFP accumulation. To summarize the above experimental results, the buckling and dome-like shape of the *Dictyostelium* lipid patch after cytosol injection occur due to the formation of lipid-protein complexes at both the outer and inner regions of the patch.

## Conclusions

The formation of the micrometer-sized lipid-protein patch from *Dictyostelium discoideum* in the current study brought us two achievements for constructing the membrane-assisted biochemical machinery. One achievement is the pre-organization of the *Dictyostelium* lipid patches. The reconstitution of the lipid-protein membrane film from *Escherichia coli* has been already established by using commercially available lipid mixtures [9]. This is because the lipid mixture extracted from *Escherichia coli* facilitates the formation of lipid membrane films supported on a substrate through the vesicle rupture method. There have been reports of lipid-protein patch formed via the break-off of living cells fixed on a substrate [42]. These lipid-protein patches have only directed the focus on the adsorbing and localizing behaviours of proteins on the lipid membrane film. Thus, such setup for research is not applicable in studying membrane film dynamics and deformation, including buckling, because the membrane film is anchored and/or fixed onto the substrate. In the current study, the spin coating method enabled us to form pre-organized lipid patches from *Dictyostelium discoideum* on the micrometer scale using a substrate holder with aspiration pores.

The other achievement is the construction of the lipid-protein membrane film, showings three-dimensional dynamic behaviours. The closed bilayer formation to prepare vesicles using natural lipids has been adopted to investigate the adsorbing and localizing behaviours of proteins. Recently, a method for preparing vesicles with diameters of larger than 1 μm, using phosphatidylcholines and phosphatidylinositides, was reported elsewhere [43]. Even though such vesicles have drawn attention in terms of artificial cell modelling, it is difficult to construct a membrane-assisted biochemical machinery to be tuned or modified inside after the encapsulation of the cytosol. The current 2D-system is one solution to this issue. Even though the thickness of the *Dictyostelium* lipid patch was evaluated to be 10.5 μm and the patch contained the stacked lipid layers, we observed the autonomous buckling of the lipid membrane film for the first time under the cytosol without any mechanical aspiration force. In the previous report [6], the membrane buckling of the *Dictyostelium* cell body itself was induced by the glass capillary aspiration and discussed in terms of the membrane bending energy and the work of the aspiration pressure. The current finding is thus crucial for the continuing development of the measurement of the cytosol ability for cell membrane deformation in an open system.

## Methods

### Cell culture

AX4 cells of the *Dictyostelium discoideum* axenic strain were cultured in modified HL5 medium under shaking at 155 rpm at 22°C [44]. The AX4 cells co-expressing the Pleckstrin homology domain of cytosolic regulator of adenylyl cyclase (PH-crac) tagged with RFP (PH-crac-RFP) and the phosphatase and tensin homolog (PTEN) tagged GFP (PTEN-GFP) were cultured in modified HL5 medium containing G418 (30 μg/mL, Wako, Japan) and Hygromycin B (60 μg/mL, Calbiochem, USA) [11].

### Cytosol extraction

Cytosolic extracts were prepared from AX4 cells according to the protocol described elsewhere [45, 46]. The cells, which were numbered at 7 × 10^6^ cells mL^-1^ were harvested, washed two times with 80 mL of phosphate buffer (PB), and re-suspended in 500 μL of PB. Cells were disrupted by nitrogen decompression using a cell disruption vessel (model 4639l, Parr Instrument, IL, USA) on ice. Cell lysate was centrifuged at 16000 × g for 30 min at 4°C. The supernatant was isolated and centrifuged again. The supernatant were transferred to a microtube and kept on ice until just before use. When we examined LY294002 as an inhibitor assay, a dimethyl sulfoxide solution (2 μL) of LY294002 (final concentration of 40 μM) was added to the cell suspension of PB (250 μL) with shaking for 30 min just before use for the extraction.

### Lipid extraction

Lipid extraction was performed according to the protocol of Bartles’s group [47] with slight modifications. AX4 cells were harvested, washed two times with 80 mL of PB, and re-suspended in phosphate buffered saline (PBS) to prepare the cell dispersion of 4.0 × 10^8^ cells mL^-1^. The dispersion was quickly frozen once in liquid nitrogen and freeze-thawed in an ice water bath. Lysates were centrifuged at 50,000 × g for 45 min at 4°C. Pellets were homogenized by a pipette in Milli-Q water. The homogenate was centrifuged again, and the washed pellets were drained and dispersed in a mixed organic solvent (CHCl_3_/CH_3_OH (2:1, v/v)). The volume of the organic solvent was 2250 μL per 4 × 10^8^ cells. The organic dispersion was centrifuged at 2000 × g for 5 min at 4°C. The organic layer was transferred to a glass bottle by an acute Pasteur pipette and the solvent was removed by nitrogen gas blowing. The residue was suspended in 938 μL of CHCl_3_/saturated NaCl aq (2:1, v/v) and the suspension was centrifuged at 2000 × g for 5 min at 4°C. The organic layer was transferred to a glass bottle by an acute Pasteur pipette.

### Spin coating and microscopy observation

On a square-type cover glass (18 × 18 mm, thickness; 0.12-0.17 mm, MATSUNAMI, Japan), we dropped 60 μL of the organic lipid solution and it was spin coated at 300 rpm for 3 min using a spin coater (MIKASA, JAPAN). As a reference experiment, a chloroform solution (60 μL) of 1-palmitoyl-2-oleoyl-3-*sn*-glycero-3-phosphocholine (POPC, 0.45 mM, Wako, Tokyo, Japan), 1,2-hexadecanoyl-3-*sn*-glycerophosphatidylinositol (3,4,5)-trisphosphate (PIP3, 10 mol%, Avanti Polar Lipids, USA), and TexasRed®-1,2-dihexadecanoyl-*sn*-glycero-3-phosphoethanolamine triethylammonium salt (TexasRed-DHPE, 0.1 mol%, Invitrogen, USA) was dropped and spin coated in the same manner. The lipid membrane film formed on the cover glass was shaded from light and set in a desiccator, and the residual organic solvent was completely removed from the lipid membrane film under reduced pressure at room temperature for 17 h. To assemble a chamber, the cover glass was attached to a perforated dish with double-sided tape (Frame Seal, thickness; 280 μm, BIO-RAD, MA, USA). The chamber was placed on a microscope stage and the lipid membrane film was pre-wetted by 50 μL of PBS for 10 min. After injection of 25 μL of the extracted cytosol, the lipid membrane film was observed under a confocal laser scanning fluorescence and bright field microscope (A1R+, Nikon, Japan) equipped with a 60× objective lens. Red and green fluorescence images were obtained through the corresponding band-pass filter and dichroic mirror units (excitation; 562 nm, emission; 570 - 620 nm and excitation; 487 nm, emission; 500 - 550 nm). The time interval for time lapse image acquisition was set to 6 s. To determine the area of lipid membrane patch formed on the cover glass, we analyzed the histogram of each Green and Red fluorescence intensity of all pixels on the captured image frame by Image J (NIH, USA) and found two peaks in the histogram. Since one peak with lower fluorescence intensity was caused by the noise from the detector and the scattered light, we measured the area of pixels where the intensity was in the range of the other peak distribution with larger intensity.

### Phalloidin staining and observation

For visualization of F-actin, we used Alexa488-labeled phalloidin (Invitrogen, USA). After injection of the cytosol has been injected for 30 min, the lipid membrane film was fixed with 3.7% formalin in PBS for 10 min. The formalin-fixed film was washed three times by 3 mL of PBS and stained by 50 μL of 5 units/mL Alexa488-labeled phalloidin dissolved in PBS for 20 min, and washed three times by PBS. The stained film was observed under a confocal laser scanning fluorescence microscope (CSU-X1, IX81, Olympus, Japan) equipped with a 100× objective lens. Red and green fluorescence images were obtained by using the corresponding band-pass filter and dichroic mirror units (excitation; 520 - 550 nm, emission; > 580 nm and excitation 470 - 490 nm, emission; 510 - 550 nm).

## Electronic supplementary material

Additional file 1: Figure S1: Bright field microscopy image of the cover glass with lipid membrane film of POPC with PIP3 (10 mol%) prepared by the spin coating. (bar = 3 mm). (DOC 5 MB)

Additional file 2: Video 1: Movie of the time-course change of *Dictyostelium* lipid patch. We captured the movie by typical bright field microscopy (time interval per frame = 6 s). The buckling motion of the lipid patch occurred remarkably for initial 10 min after AX4 cytosol injection (24 – 30 s). The size of the observation space is 210 μm x 210 μm. (ZIP 6 MB)

Additional file 3: Video 2: Movie of the time-course change of *Dictyostelium* lipid patch. We captured the movie by the confocal laser scanning fluorescence microscopy with both Red and Green detection modes (time interval per frame = 6 s). The PTEN-GFP and PH-crac-RFP localization on the *Dictyostelium* lipid patch occurred after injection of cytosol extract of PH-crac-RFP/PTEN-GFP co-expressing cells (24 – 30 s). The size of the observation space is 210 μm x 210 μm. (ZIP 5 MB)

Additional file 4: Video 3: Movie of the time-course change of *Dictyostelium* lipid patch. We captured the movie by the typical bright field microscopy (time interval per frame = 6 s). The buckling motion occurred remarkably for initial 10 min after injection of cytosol extract of PH-crac-RFP/PTEN-GFP co-expressing cells (24 – 30 s). The size of the observation space is 210 μm x 210 μm. (ZIP 6 MB)

Additional file 5: Figure S2: 3D-merged reconstructed images of the autofluorescence images of a typical *Dictyostelium* lipid patch obtained by confocal laser scanning fluorescence microscopy with both Red and Green detection modes before (A,B) and 30 min after (C,D) injection of cytosol extract, which had been treated at 65°C for 1 h just before use, of PTEN-GFP/PH-crac-RFP co-expressing cells. The volume size of observation space is 210 μm × 210 μm × 37.5 μm. Each x-z plane cross-section of the 3D merged reconstructed image was attached in the right column (B,D). Dashed lines correspond to the surface of the glass slide. The height indicates the distance between the surface of the glass slide and the peak top position of the autofluorescence images. (DOC 797 KB)

## Abbreviations

POPC: 1-palmitoyl-2-oleoyl-3-*sn*-glycero-3-phosphocholine
PIP3: 1,2-hexadecanoyl-3-*sn*-glycerophosphatidyl inositol-(3,4,5)-trisphosphate
PTEN: Phosphatase and tensin homolog
PTEN-GFP: PTEN tagged green fluorescent protein
PH-crac: Pleckstrin homology domain of cytosolic regulator of adenylyl cyclase
PH-crac-RFP: PH-crac tagged with RFP.
