# Supplementary figures and images for: Autonomous buckling of micrometer-sized lipid-protein membrane patches constructed by Dictyostelium discoideum
Source: J Biol Eng. 2015 Jan 21;9:3. doi: 10.1186/1754-1611-9-3 (PMC4429478; doi:10.1186/1754-1611-9-3)

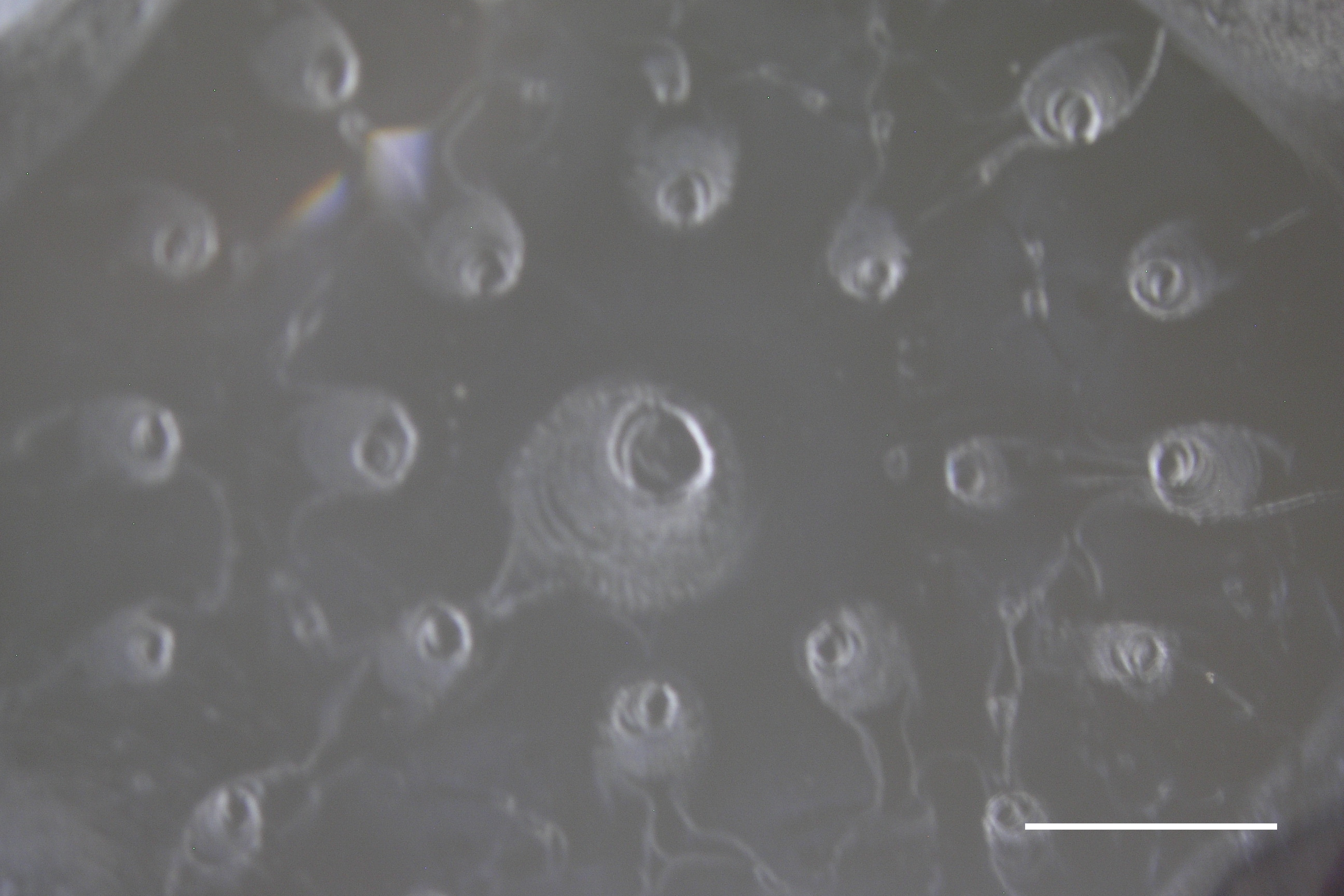


**Figure Supplement 1.**

Kei Takahashi et al.

Supplement: Supplementary file 1 — Additional file 1: Figure S1: Bright field microscopy image of the cover glass with lipid membrane film of POPC with PIP3 (10 mol%) prepared by the spin coating. (bar = 3 mm). (DOC 5 MB) [file 13036_2014_165_MOESM1_ESM.doc]

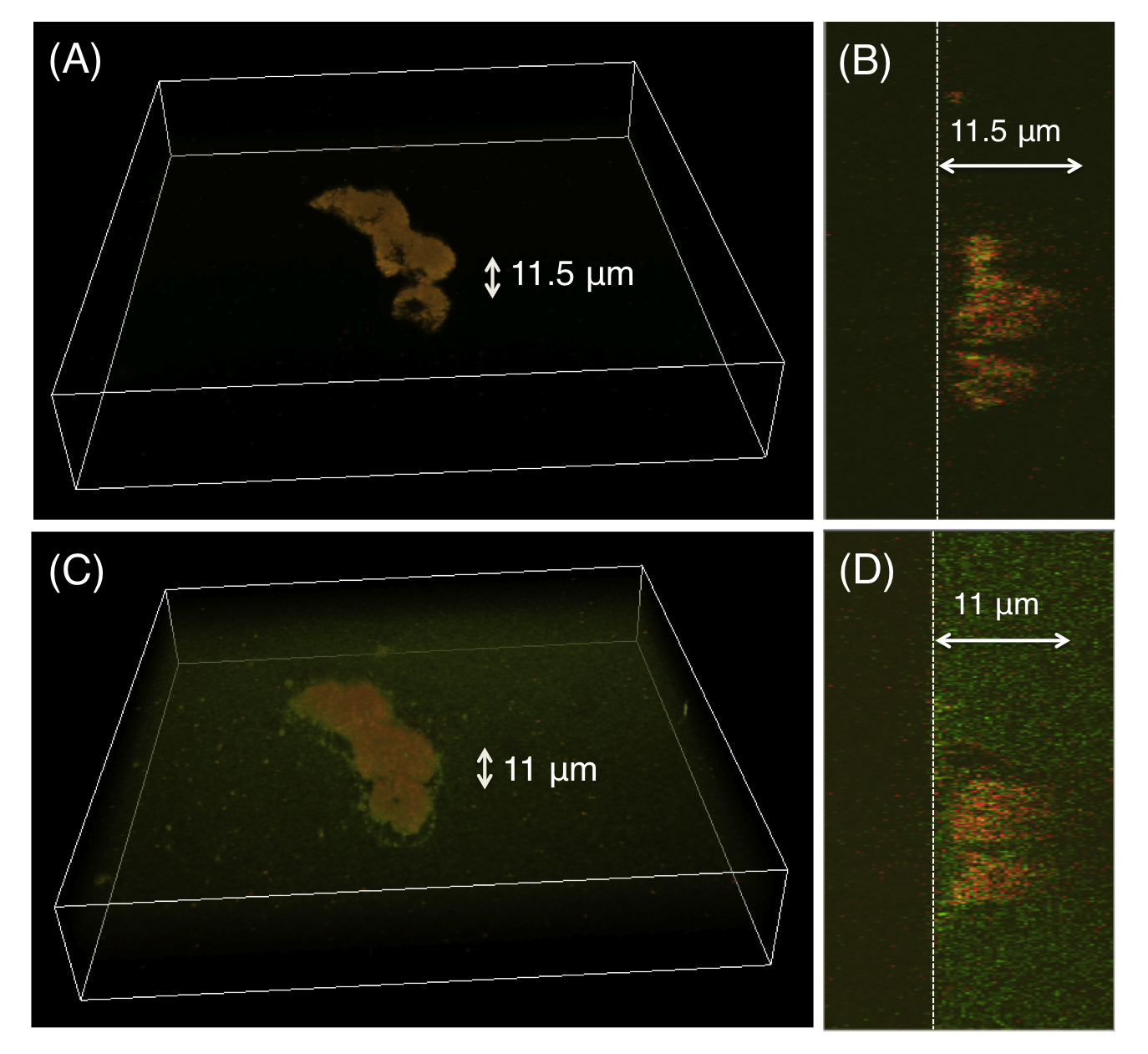


**Figure Supplement 2.**

Kei Takahashi et al.

Supplement: Supplementary file 5 — Additional file 5: Figure S2: 3D-merged reconstructed images of the autofluorescence images of a typical Dictyostelium lipid patch obtained by confocal laser scanning fluorescence microscopy with both Red and Green detection modes before (A,B) and 30 min after (C,D) injection of cytosol extract, which had been treated at 65°C for 1 h just before use, of PTEN-GFP/PH-crac-RFP co-expressing cells. The volume size of observation space is 210 μm × 210 μm × 37.5 μm. Each x-z plane cross-section of the 3D merged reconstructed image was attached in the right column (B,D). Dashed lines correspond to the surface of the glass slide. The height indicates the distance between the surface of the glass slide and the peak top position of the autofluorescence images. (DOC 797 KB) [file 13036_2014_165_MOESM5_ESM.doc]
